# Optimal Antithrombotic Regimens Across Atherosclerotic Vascular Beds: Toward Mechanism and Risk-Oriented Strategies

**DOI:** 10.3390/jcm15062325

**Published:** 2026-03-18

**Authors:** Pierre Sabouret, Domenico Mario Giamundo, Francesco Costa, Piera Capranzano, Luigi Spadafora, Stefano Cacciatore, Nelsa González Aguado, Marco Bernardi, Ahmed Ibrahim, Ali Abdelaziz, Giulia Alagna, Felice Gragnano, Paolo Calabrò, Giuseppe Andò

**Affiliations:** 1 Heart Institute and Action Group, Pitié-Salpétrière, Sorbonne University, 75013 Paris, France; 2 Department of Cardiology, Policlinico Casilino, 00168 Rome, Italy; jamundus20@libero.it; 3 Department of Biomedical and Dental Sciences and of Morphological and Functional Images, University of Messina, 98122 Messina, Italy; 4 Cardiology Division, Policlinico Hospital, University of Catania, 95124 Catania, Italy; 5 Department of Medical-Surgical Sciences and Biotechnologies, Sapienza University of Rome, 04100 Latina, Italy; 6 Department of Geriatrics, Orthopedics and Rheumatology, Università Cattolica del Sacro Cuore, 00168 Rome, Italy; 7 Fondazione Policlinico Universitario “Agostino Gemelli” IRCCS, 00168 Rome, Italy; 8 Área del Corazón, Hospital Universitario Virgen de La Victoria, 29010 Málaga, Spain; 9 Unidad de Insuficiencia Cardíaca y Cardiopatías Familiares, Cardiología, Hospital Universitario Virgen de La Victoria, 29010 Málaga, Spain; 10 Department of Cardiology, Santa Maria Goretti, 04100 Latina, Italy; 11 Faculty of Medicine, Alexandria University, Alexandria 5424041, Egypt; 12 Cardiology Department, San Biagio Hospital, ASL VCO Distretto Sanitario di Verbania, 28922 Verbania, Italy; 13 Department of Translational Medical Sciences, University of Campania “Luigi Vanvitelli”, 80138 Naples, Italy; 14 Division of Cardiology, A.O.R.N. “Sant’Anna e San Sebastiano”, 26100 Caserta, Italy; 15 Azienda Ospedaliera Papardo, Contrada Papardo, 98158 Messina, Italy; 16 Department of Clinical and Experimental Medicine, University of Messina, 98122 Messina, Italy

**Keywords:** antithrombotic therapy, atherosclerosis, vascular disease, antiplatelet, anticoagulant, precision medicine

## Abstract

Arterial thrombosis emerges from the interplay between plaque disruption, platelet activation, and coagulation pathway amplification on a background of heterogeneous ischemic and bleeding risk. Optimal antithrombotic therapy therefore varies across clinical settings, from acute coronary syndromes (ACS) to chronic coronary syndromes (CCS), ischemic stroke, peripheral artery disease (PAD), and atrial fibrillation (AF) associated with atherosclerotic disease. Contemporary European and North American guidelines endorse an increasingly individualized approach, moving away from rigid “one-size-fits-all” dual antiplatelet therapy (DAPT) duration and intensity and incorporating dual pathway inhibition with low-dose rivaroxaban plus aspirin in selected high-risk CCS and PAD patients. In ischemic stroke, short-course DAPT is confined to minor events and transient ischemic attacks, whereas long-term monotherapy remains standard, and the coexistence of AF typically shifts the balance toward oral anticoagulation. Across all scenarios, antithrombotic benefit must be weighed against bleeding, especially in elderly, frail, or comorbid patients. Evidence gaps remain substantial, particularly in patients with overlapping vascular territories, AF plus atherosclerotic disease, and after ischemic stroke of complex or mixed mechanisms. This narrative review summarizes current evidence and guideline-based strategies in major atherosclerotic settings, proposes a unifying conceptual framework, and highlights key uncertainties and research directions for truly personalized antithrombotic care.

## 1. Introduction

Plaque rupture or erosion triggers platelet adhesion, activation, and aggregation, together with tissue factor–mediated thrombin generation that stabilizes the thrombus and propagates occlusion; both arms of thrombosis therefore represent therapeutic targets [1].

Over recent decades, increasingly potent antiplatelet and anticoagulant strategies have reduced ischemic events, but at the price of higher major bleeding, including intracranial hemorrhage [2]. The clinical challenge is to identify for each patient the minimum intensity and duration of antithrombotic therapy that maintains effective prevention of recurrent atherothrombosis or cardioembolism without disproportionate bleeding risk. Several concepts support a setting-specific approach. First, the relative contribution of platelet-driven versus coagulation-driven thrombosis differs between acute coronary syndrome (ACS), chronic coronary disease, non-cardioembolic stroke, peripheral artery disease (PAD), and atrial fibrillation (AF)-related cardioembolism.

Second, the delicate balance between ischemic and bleeding risk is not static but evolves. The risk of recurrent atherothrombotic events is highest in the acute phase following an ACS or stroke and gradually declines thereafter. In contrast, the risk of bleeding, particularly with prolonged combination therapy, tends to persist or even accumulate.

Third, individual patient phenotypes, defined by age, comorbidities (e.g., renal dysfunction, diabetes), and concomitant therapies (e.g., the need for chronic anticoagulation in AF), profoundly modify the therapeutic window for any given drug combination. These converging concepts have collectively driven a decisive shift in contemporary guidelines, moving from “one-size-fits-all” algorithms toward individualized regimens based on structured risk assessment, shared decision-making, and flexible algorithms for de-escalation or intensification [2,3,4,5,6]. Furthermore, growing recognition of the heterogeneity of thrombotic mechanisms in different vascular beds has challenged traditional uniform therapeutic approaches, highlighting the need for a more mechanistic and patient-centered framework that integrates clinical presentation, comorbidities, and dynamic risk profiles [7,8]. Most available reviews on antithrombotic therapy focus on individual vascular territories or summarize guideline recommendations within a single clinical setting. In contrast, the aim of the present review is to integrate current evidence across the major atherosclerotic vascular beds within a unified conceptual framework. By linking dominant thrombotic mechanisms, temporal phases of disease, and individual ischemic and bleeding risk profiles, this approach seeks to provide a more pragmatic and clinically oriented perspective to guide personalized antithrombotic strategies in everyday practice.

## 2. Methods

This narrative review was conducted to summarize the current evidence on antithrombotic strategies in major atherosclerotic vascular beds, including acute coronary syndromes, chronic coronary syndromes, ischemic stroke, peripheral artery disease, and atrial fibrillation with coexisting atherosclerosis. A comprehensive literature search was performed using the PubMed/MEDLINE, Scopus, and Web of Science databases for studies published between January 2015 and December 2025. The search strategy combined Medical Subject Headings (MeSH) and free-text terms including “antithrombotic therapy”, “dual antiplatelet therapy”, “dual pathway inhibition”, “rivaroxaban”, “atherosclerosis”, “acute coronary syndrome”, “chronic coronary syndrome”, “ischemic stroke”, “peripheral arterial disease” and “atrial fibrillation”. Priority was given to randomized controlled trials, meta-analyses and contemporary international guidelines from major scientific societies, including the European Society of Cardiology (ESC), the American College of Cardiology (ACC) and the American Heart Association (AHA). Additional relevant studies were identified through manual selection of the reference lists of selected articles. As this is a narrative review, no formal systematic selection process or quantitative synthesis was performed. However, emphasis was placed on high-quality evidence and clinically relevant data to provide a pragmatic and up-to-date overview of mechanism- and risk-oriented antithrombotic strategies. Preference was given to randomized controlled trials, meta-analyses, and the most recent international guideline documents. Earlier landmark trials were retained when considered essential to explain the development of current therapeutic strategies. When multiple studies addressed similar questions, priority was given to larger randomized datasets and guideline-defining trials. In the presence of apparently conflicting findings, interpretation was guided by differences in study populations, clinical settings, and bleeding-risk profiles rather than by isolated efficacy signals.

## 3. Pathophysiological Determinants of Thrombosis in Vascular Beds

Arterial thrombosis is a dynamic process, resulting from the interaction between vascular wall injury, platelet activation, and activation of the coagulation cascade. However, the relative contribution of these mechanisms varies considerably across different vascular territories and clinical scenarios, providing the biological justification for personalized antithrombotic strategies. In acute coronary syndromes, plaque rupture or erosion exposes highly thrombogenic material, including tissue factor and collagen, triggering intense platelet adhesion and aggregation along with rapid thrombin generation. This dual activation explains the central role of dual antiplatelet therapy, often supplemented by transient anticoagulation in the acute phase [7,8]. In chronic coronary syndromes, the pathophysiological substrate is more heterogeneous, characterized by stable plaques, endothelial dysfunction and low-grade thrombin generation. In this context, both platelet activation and coagulation pathways contribute to recurrent events, supporting the concept of dual pathway inhibition with low-dose factor Xa inhibition plus aspirin in selected high-risk patients [3,4]. Ischemic stroke involves a number of mechanisms, including large artery atherosclerosis, small vessel disease, and cardioembolism. In non-cardioembolic stroke, platelet activation plays a predominant role, justifying antiplatelet therapy as a cornerstone of secondary prevention. In contrast, in cardioembolic stroke related to atrial fibrillation, thrombin generation in the left atrium prevails, making oral anticoagulant therapy the most effective preventive strategy [9,10]. Peripheral arterial disease represents a widespread atherothrombotic condition with a particularly high burden of systemic inflammation and thrombin generation. Patients with PAD show both increased platelet reactivity and activation of the coagulation cascade, which may explain the remarkable benefit observed with dual pathway inhibition in reducing cardiovascular and limb events [11]. Finally, in patients with atrial fibrillation and concomitant atherosclerotic disease, the thrombotic risk arises from overlapping mechanisms. The cardioembolic risk caused by atrial blood stasis coexists with platelet-mediated atherothrombosis, creating a complex therapeutic scenario in which the combination and duration of antiplatelet and anticoagulant therapies must be carefully balanced to optimize the net clinical benefit [12,13]. Overall, these mechanistic differences highlight that antithrombotic therapy should not be uniform across all vascular beds but rather aligned with the dominant biological factors of thrombosis in each clinical setting. In support of the main antithrombotic strategies in different clinical settings, the main randomized studies are summarized in Table 1.

## 4. Optimal Antithrombotic Strategy in Acute Coronary Syndromes

### 4.1. Early and Short-Term Phase

ACS remains the archetype of platelet-driven thrombosis, where immediate stabilization of the disrupted plaque and prevention of stent-related complications are paramount. Consequently, potent dual antiplatelet therapy (DAPT) is the cornerstone of early management. The 2023 ESC ACS and 2025 ACC/AHA guidelines reaffirm 12-month DAPT with aspirin plus a potent P2Y12 inhibitor (ticagrelor or prasugrel) as the default regimen in patients without high bleeding risk (HBR), irrespective of whether they have ST-segment elevation or non-ST-segment elevation ACS [2,22]. In practical terms, the default DAPT duration after ACS is 12 months in patients without high bleeding risk, irrespective of whether the presentation is ST-segment elevation or non-ST-segment elevation ACS. This strategy is indicated to reduce recurrent myocardial infarction, stent thrombosis, and cardiovascular death, particularly after PCI and in patients with a clear plaque-related thrombotic mechanism. In contrast, DAPT should not be considered a uniform requirement in all patients, and its duration should be adapted when bleeding risk predominates [2,22]. This strategy is supported by a broad trial portfolio (e.g., PLATO [14], TRITON-TIMI 38 [15]) demonstrating substantial reductions in recurrent myocardial infarction, stent thrombosis, and cardiovascular death compared with clopidogrel-based or shorter DAPT [23].

Intravenous glycoprotein IIb/IIIa inhibitors, once widely used in the management of acute coronary syndromes, currently have a much more limited role in contemporary practice. With the widespread use of potent oral P2Y12 inhibitors and improved PCI techniques, their routine upstream administration is no longer recommended. However, they may still be considered as bailout therapy during PCI in selected situations such as large thrombus burden, slow or no-reflow, or other thrombotic complications [2].

However, guideline recommendations now consider nuanced pathways allowing shortened DAPT (3–6 months) in patients at HBR or who are event-free with low ischemic risk, as supported by multiple randomized trials, mainly TWILIGHT [16] and TICO [17] trials, and meta-analyses of abbreviated DAPT [23]. At the same time, it is increasingly recognized that the response to antiplatelet agents, particularly clopidogrel, varies greatly between individuals, depending on comorbidities, drug interactions and genetic determinants. In particular, CYP2C19 polymorphisms have been associated with significant differences in active metabolite activity and clinical outcomes, contributing to the debate on personalized (genotype-guided) or platelet function testing-guided strategies [24,25]. In this context, the TROPICAL-ACS trial showed that platelet testing-guided de-escalation (early switch from prasugrel to clopidogrel) may be a viable alternative in selected patients, maintaining a net clinical benefit that is no less than that of a more intensive standard strategy [25]. Similarly, pharmacogenomic approaches have been explored in patients with ACS, suggesting that personalized selection of P2Y12 inhibitors could reduce ischemic events and/or bleeding in specific subpopulations, although questions remain about feasibility and large-scale implementation [24]. In patients with increased bleeding liability, clopidogrel remains the preferred P2Y12 inhibitor when a less intensive strategy is required, particularly in the context of de-escalation strategies or when combination therapy with oral anticoagulation is necessary. Options include early transition to P2Y12 inhibitor monotherapy after 1–3 months, particularly with ticagrelor or, in patients who develop bleeding complications, are at HBR, or have tolerability issues (e.g., nuisance bleeding), an early switch from a potent P2Y12 inhibitor to a less potent, clopidogrel-based DAPT. However, the recommendation for early DAPT de-escalation at 1 month in HBR patients remains weak (Class IIb, Level B). This clinical uncertainty is further highlighted by the HOST-BR trial [26], which indicated that 1-month DAPT failed to be non-inferior to 3 months regarding ischemic protection. While the use of risk scores for ischemia and bleeding is encouraged, they remain suboptimal, and clinician judgment remains crucial with shared decision-making to balance the fine line between thrombotic prevention and hemorrhagic safety. Further evidence concerns the early discontinuation of aspirin and continuation of P2Y12 inhibitor therapy alone after a short period of DAPT (‘P2Y12 inhibitor monotherapy’), with the aim of reducing the risk of bleeding while maintaining acceptable antithrombotic efficacy. Randomized studies and subsequent analyses have supported the plausibility of this strategy in selected populations undergoing PCI, with a consistent reduction in bleeding and an overall neutral signal on ischemic endpoints [27,28]. However, the results are not uniform in all contexts: in “pure” and high-risk ACS populations, excessively early reduction in antithrombotic intensity may be problematic. In particular, in the STOPDAPT-2 ACS trial, the strategy of clopidogrel monotherapy after 1–2 months did not demonstrate non-inferiority compared with standard DAPT, with a reduction in bleeding but a numerical signal of increased cardiovascular events [29]. These data reinforce the need to carefully select patients who are candidates for early monotherapy strategies, integrating anatomical/procedural risk and overall clinical profile. In particular, real-world registries and observational studies have consistently shown that hemorrhagic events are not only frequent but also independently associated with subsequent mortality, reinforcing the concept that hemorrhage prevention is a key therapeutic goal alongside ischemia prevention [30,31,32].

### 4.2. Long-Term Phase After ACS (≥12 Months)

Beyond 12 months post-ACS, aspirin monotherapy is recommended as the standard long-term antithrombotic agent by ESC guidelines (Class I, Level A), irrespective of bleeding risk category [2]. In this context, prolonged DAPT refers to continuation beyond 12 months after the index ACS or PCI [2,33] and may be considered in selected patients with high ischemic risk and no high bleeding risk, such as those with prior myocardial infarction, diffuse multivessel disease, recurrent ischemic events, diabetes, chronic kidney disease, or polyvascular disease [34]. However, the expected absolute ischemic benefit should be carefully weighed against the cumulative risk of major bleeding. If bleeding complications occur, or if the patient evolves toward a high bleeding risk phenotype, treatment simplification should be promptly considered, including discontinuation of one antiplatelet agent, transition to single antiplatelet therapy, or avoidance of further intensified strategies. Selected very high ischemic risk patients without HBR (e.g., diffuse multivessel disease, recurrent events, polyarterial disease) may benefit from extended intensified strategies such as aspirin plus ticagrelor 60 mg twice daily [35], or dual pathway inhibition with aspirin plus low-dose rivaroxaban, extrapolating from COMPASS and other datasets [1]. Conversely, de-escalation to clopidogrel or even P2Y12 monotherapy may be appropriate in certain populations, reflecting the paradigm that “less is more” when bleeding risk overcomes ischemic recurrence. However, identifying patients who derive a net benefit from prolonged intensified therapy remains difficult, as commonly used risk scores show only modest discrimination and limited applicability in different clinical settings [36]. From an operational standpoint, the use of dedicated tools can nevertheless support clinical discussion when evaluating prolonged or intensified therapy. PRECISE-DAPT was developed to estimate the risk of out-of-hospital bleeding during DAPT and may be useful in identifying patients in whom early simplification is reasonable [37]. In contrast, the DAPT score aims to balance the potential ischemic benefit and bleeding risk of continuing DAPT beyond one year after PCI, helping to identify a subgroup with a higher probability of net benefit [38]. In addition, the ARC-HBR definition has provided pragmatic and standardized criteria for identifying patients at high risk of bleeding, improving comparability between studies and supporting more consistent decisions in clinical practice [39]. Overall, these tools do not replace clinical judgment, but they can make patient selection more structured when the margins of net benefit are small.

### 4.3. Optimal Antithrombotic Treatment in Chronic Coronary Syndromes (CCS)

In CCS, single antiplatelet therapy with aspirin remains the backbone for most patients with established coronary artery disease [6]. However, this conventional approach is currently evolving as clopidogrel monotherapy is increasingly recognized as a valid and potentially superior alternative, particularly in aspirin intolerance or in light of data suggesting a modest advantage in some contexts [40]. This shift is supported by high-certainty evidence from a recent individual patient data meta-analysis, which challenged the long-standing aspirin default by demonstrating that clopidogrel monotherapy is superior to aspirin for reducing major adverse cardiovascular and cerebrovascular events (MACCE) without increasing major bleeding in patients with established CAD [41]. These findings have reignited the debate on the optimal first-line antiplatelet agent in stable coronary artery disease, particularly in light of interindividual variability in response to aspirin and the emerging concept of personalized antiplatelet strategies. The central question in CCS is whether selected high-risk patients should receive intensified therapy beyond standard single antiplatelet treatment (SAPT).

The investigators of the COMPASS trial reported that low-dose rivaroxaban 2.5 mg twice daily plus aspirin (dual pathway inhibition) reduces major adverse cardiovascular events (MACE) compared with aspirin alone in patients with stable coronary or peripheral artery disease, at the cost of increased major bleeding but with no excess in fatal or intracranial hemorrhage [1]. Subsequent analyses suggest that patients with polyvascular disease, diabetes, or renal dysfunction derive the greatest absolute benefit, supporting a targeted use of dual pathway inhibition as a vascular protection strategy [42]. From a practical standpoint, the patients most likely to derive meaningful absolute benefit from dual pathway inhibition are those with diffuse or polyvascular atherosclerotic disease, particularly when diabetes, renal dysfunction, or recurrent ischemic events coexist and bleeding risk remains acceptable. In fact, the presence of polyvascular disease is one of the strongest determinants of absolute ischemic risk, with event rates increasing progressively as the number of affected areas increases. Evidence from secondary prevention registries and cohorts has documented that patients with concomitant coronary, peripheral and/or cerebrovascular involvement have a significantly worse prognosis than those with single-district disease, making the concept of absolute (as well as relative) benefit particularly relevant when considering intensified strategies [43,44]. At the same time, the issue of residual risk not fully explained by thrombosis alone is emerging strongly in CCS: vascular inflammation contributes to plaque vulnerability and event recurrence even in the presence of optimal control of traditional risk factors. Some exploratory studies have also suggested that dual pathway inhibition may be associated with modest reductions in selected inflammatory biomarkers, although the clinical significance of these observations remains uncertain and requires further investigation [45]. Anti-inflammatory therapy trials have provided clinical proof of concept for the inflammatory hypothesis of atherothrombosis, paving the way for integrated strategies (antithrombotic + inflammation modulation) in selected patients [46]. Consistently, even drugs with more “accessible” anti-inflammatory activity, such as low-dose colchicine, have shown a reduction in cardiovascular events in patients with chronic coronary artery disease, suggesting that vascular protection in patients at high residual risk may require a multimodal approach [47]. In contrast, full-dose oral anticoagulation is not recommended purely for atherothrombotic prevention in CCS, in the absence of another indication such as AF or venous thromboembolism [6]. Another mechanistically attractive strategy is thrombin receptor antagonism through inhibition of the protease-activated receptor-1 (PAR-1). Vorapaxar has been shown to reduce recurrent ischemic events in selected patients with prior myocardial infarction or peripheral artery disease. However, this benefit was accompanied by a significant increase in bleeding, particularly intracranial hemorrhage in patients with previous stroke, which has limited its adoption in routine clinical practice [48].

Guidelines, therefore, promote individualized selection between:

- Aspirin (or clopidogrel) monotherapy for the majority, with recent RCTs advocating a change in paradigm due to the more pronounced CV benefits with clopidogrel monotherapy, even if the absolute risk reduction remains debatable [40].
- Dual pathway inhibition (rivaroxaban 2.5 mg twice daily plus aspirin) in high ischemic/low bleeding risk patients, a rarer situation due to the frequent overlap between ischemic and bleeding risks (age, chronic kidney disease, etc.) [1].
- In a minority, prolonged DAPT after prior ACS or percutaneous coronary intervention (PCI) where ischemic risk remains high due to suboptimal RF control and/or coronary anatomy associated with an acceptable bleeding risk [2].

In patients with CCS undergoing PCI, the default strategy post-PCI is DAPT (aspirin 75–100 mg and clopidogrel 75 mg) for 6 months (Class I, Level A). In those at high bleeding risk but not high ischemic risk, DAPT should be discontinued after 1–3 months in favor of SAPT (Class I, Level A). Stopping DAPT after 1–3 months may also be considered in patients who are at neither high bleeding nor high ischemic risk (Class IIb, Level B). Finally, for high-thrombotic-risk stenting, including complex left main stem, 2-stent bifurcation, suboptimal results, or prior stent thrombosis, prasugrel or ticagrelor may be considered instead of clopidogrel for the first 1–6 months (Class IIb, Level C) [49]. A further element of complexity in the management of CCS is represented by the difficulty of translating evidence from randomized trials into everyday clinical practice. Patients enrolled in clinical trials are often more selected, with less comorbidity and a lower risk of bleeding than the real population, in which the coexistence of multiple risk factors makes therapeutic choice more complex. In this context, data from observational registries suggest that intensified antithrombotic strategies, while effective in reducing ischemic events, are frequently underused in high-risk patients, while they may be overused in subjects with high bleeding risk [50]. This “therapeutic paradox” highlights the need to improve the implementation of guidelines and to develop more accurate tools for risk stratification in clinical practice.

### 4.4. Optimal Antithrombotic Regimen in Ischemic Atherosclerotic Stroke

In non-cardioembolic ischemic stroke or transient ischemic attack (TIA), antiplatelet therapy (APT) is the default secondary prevention strategy [4]. Current ACC and stroke society key points advise initiation of an antiplatelet agent (aspirin, clopidogrel, or aspirin–dipyridamole) within 24–48 h of symptom onset, once hemorrhage has been excluded [4]. For minor ischemic stroke or high-risk TIA, DAPT with aspirin plus clopidogrel or ticagrelor is recommended for a short period (~21 days), followed by long-term single antiplatelet therapy (SAPT), reflecting evidence that early DAPT reduces recurrent ischemic events. Still, prolonged DAPT increases major bleeding without additional ischemic benefit [42,51,52].

Patients with embolic stroke of undetermined source exemplify the limits of empirical oral anticoagulation (OAC). Indeed, a large RCT of DOACs versus aspirin failed to demonstrate superiority of anticoagulation [18,19], and current guidance favors SAPT in this group [4]. In specialized situations such as antiphospholipid antibody syndrome or cancer-associated stroke, anticoagulation is usually preferred, underscoring the importance of mechanism-directed therapy. Overall, long-term DAPT has a very limited role in atherosclerotic stroke prevention, and there is currently no guideline-endorsed role for low-dose rivaroxaban plus aspirin in this setting outside of coexisting PAD or CCS, although a mechanistic rationale exists [4]. Furthermore, the heterogeneity of stroke mechanisms highlights the limitations of a “universal” approach, emphasizing the need for improved etiological classification and biomarker-guided strategies to refine secondary prevention. An additional element that is often underestimated in post-stroke secondary prevention is adherence to therapy over time. Studies based on administrative data and real-world registries have shown that early discontinuation or non-persistence with antiplatelet therapy in the months following an ischemic event is not uncommon, especially in elderly patients and those with comorbidities, and is associated with worsening long-term outcomes [53,54]. Therefore, these data support the need to complement pharmacological choices with a structured focus on implementation (education, follow-up, simplification of regimens where possible) in order to maximize the real effectiveness of the strategies recommended by the guidelines.

### 4.5. Optimal Antithrombotic Regimen in Peripheral Artery Disease (PAD)

Symptomatic PAD is associated with the highest systemic atherothrombotic risk, including myocardial infarction (MI) and stroke, as demonstrated in the worldwide REACH registry [3]. Traditionally, SAPT with aspirin or clopidogrel has been the standard of care for systemic and limb event prevention, with an apparent superiority of clopidogrel (CAPRIE study) [40]. The COMPASS trial established that rivaroxaban 2.5 mg twice daily plus aspirin reduces MACE and major adverse limb events (MALE) in patients with stable PAD, especially those with polyvascular disease, although at the cost of increased major bleeding [1]. The antithrombotic should be associated with an intensive lipid-lowering strategy, which provides additional benefits in this very high-risk population. In this context, the most recent evidence indicates that intensive LDL cholesterol reduction not only translates into a decrease in major cardiovascular events, but may also influence peripheral outcomes. In a dedicated analysis of the FOURIER trial, PCSK9 inhibition with evolocumab in patients with PAD was associated with a reduction in cardiovascular events and a benefit on major adverse limb events, supporting the idea of “pan-vascular” protection that complements antithrombotic therapy and aggressive risk factor optimization [55]. This is particularly relevant in patients with PAD and multivascular disease, where high absolute risk amplifies the potential benefit of combined strategies, provided that the hemorrhagic profile remains acceptable.

The VOYAGER PAD trial extended this concept to patients undergoing lower-extremity revascularization, showing that rivaroxaban 2.5 mg twice daily plus aspirin reduces acute limb ischemia, major amputation, myocardial infarction, ischemic stroke, and cardiovascular death versus aspirin alone, again with a bleeding trade-off but favorable net clinical benefit [56]. These data underpin contemporary recommendations that, in symptomatic PAD (particularly after revascularization), dual pathway inhibition should be considered in patients with high ischemic and limb risk and acceptable bleeding risk, while SAPT remains appropriate for lower-risk individuals with HBR [3]. It is important to emphasize that, despite their high cardiovascular risk, patients with PAD often receive insufficient treatment in clinical practice, suggesting a substantial gap between the recommendations provided by guidelines and their application in real life.

### 4.6. Optimal Antithrombotic Therapy in Atrial Fibrillation with Associated Atherosclerotic Events

The coexistence of AF and atherosclerotic disease (ACS, CCS, or PAD) poses a particular challenge because cardioembolic and atherothrombotic risks coexist and often require both oral anticoagulation and antiplatelet therapy. Triple antithrombotic therapy is primarily indicated in patients with atrial fibrillation who present with acute coronary syndrome and undergo percutaneous coronary intervention, a clinical scenario in which both cardioembolic risk and early stent-related thrombotic risk must be simultaneously addressed. For AF patients with ACS undergoing percutaneous coronary intervention, the 2023 ESC ACS guidelines and expert consensus documents recommend a short period (up to 1 week) of triple antithrombotic therapy (oral anticoagulant, aspirin, and clopidogrel), followed by dual therapy (non-vitamin K antagonist oral anticoagulant plus clopidogrel) for up to 12 months, and then oral anticoagulant monotherapy thereafter in most patients [2,3,4,5,6]. This strategy balances the high early risk of stent thrombosis and recurrent ACS against the substantial bleeding risk of prolonged triple therapy, as shown in multiple AF-PCI trials [20,21,57]. When combination therapy is required, careful attention to appropriate DOAC dosing is essential, as both unjustified dose reduction and unnecessary continuation of antiplatelet therapy may negatively affect the balance between ischemic protection and bleeding risk. It should be noted that the majority of randomized evidence supporting these strategies derives from coronary populations undergoing PCI. Consequently, recommendations for patients with atrial fibrillation and predominantly peripheral or cerebrovascular atherosclerotic disease are often extrapolated from coronary trials and should be interpreted with appropriate clinical judgment. In stable CCS or PAD with AF, long-term therapy is generally based on a DOAC (or warfarin when indicated), with the addition of a single antiplatelet agent only in selected patients with very high ischemic risk, recent revascularization, or recurrent events [5]. The evidence supporting OAC monotherapy, primarily with DOACs, as the default strategy is further reinforced by a recent study-level meta-analysis, which demonstrated that OAC alone significantly reduces major bleeding and improves net adverse clinical outcomes without increasing ischemic risk compared to combination therapy [58]. A particularly important aspect in the management of patients with AF and concomitant atherosclerotic disease is the significant heterogeneity of thrombotic and hemorrhagic risk over time. In particular, ischemic risk tends to be concentrated in the early stages after acute coronary syndrome or revascularization, while bleeding risk persists in the long term and may increase progressively with prolonged exposure to combination therapies. This divergent temporal pattern has led to the development of time-dependent strategies, in which a more intensive initial phase (triple or dual therapy) is followed by progressive simplification to anticoagulant monotherapy in most patients. However, in clinical practice, the optimal duration of the different phases remains a subject of debate, especially in patients at high ischemic risk, such as those with complex coronary anatomy or polyvascular disease. Furthermore, the management of these patients is further complicated by the presence of clinical factors that simultaneously increase ischemic and hemorrhagic risk, such as advanced age, frailty and renal dysfunction. This phenomenon of “risk overlap” represents one of the main challenges in contemporary antithrombotic medicine and limits the applicability of standardized approaches, reinforcing the need for individualized and dynamic patient assessment [47]. Routine combination of low-dose rivaroxaban (2.5 mg twice daily) plus aspirin is not applicable in patients who already require full-dose anticoagulation for AF, as this strategy has only been validated in patients without an indication for therapeutic anticoagulation [5]. After ischemic stroke in the context of AF, antithrombotic management is dominated by anticoagulation; antiplatelet agents are usually discontinued unless there is a compelling additional indication, such as recent coronary stenting, given the excess bleeding with combined therapy [5]. The recent ATIS-NVAF trial further supports this approach, demonstrating that in patients with ischemic stroke, AF, and atherosclerotic cardiovascular disease, adding an antiplatelet agent to anticoagulation does not reduce ischemic events but significantly increases bleeding compared to anticoagulant monotherapy [59]. Timing of anticoagulation initiation post-stroke is tailored to infarct size, hemorrhagic transformation, and individual risk factors, but the principle that OAC is the cornerstone remains consistent. This delicate balance is further complicated by the progressive increase in bleeding risk over time, which often leads to premature discontinuation of combination therapies in clinical practice.

### 4.7. Overview: Converging Principles Across Vascular Beds

Across ACS, CCS, stroke, PAD, and AF-associated atherosclerotic disease, several main principles have emerged. First, antithrombotic therapy must be tailored to the dominant mechanism of events: platelet-mediated thrombosis in ACS and PAD, combined platelet and coagulation activation in CCS and polyvascular disease, and primarily cardioembolic mechanisms in AF. Second, early phases following an acute event or revascularization justify transient intensification (potent DAPT or short-term triple therapy, or short-course DAPT after minor stroke/TIA), whereas long-term prevention favors simplified regimens (SAPT, dual pathway inhibition in selected patients, or OAC alone) [2,4].

**Figure 1:**
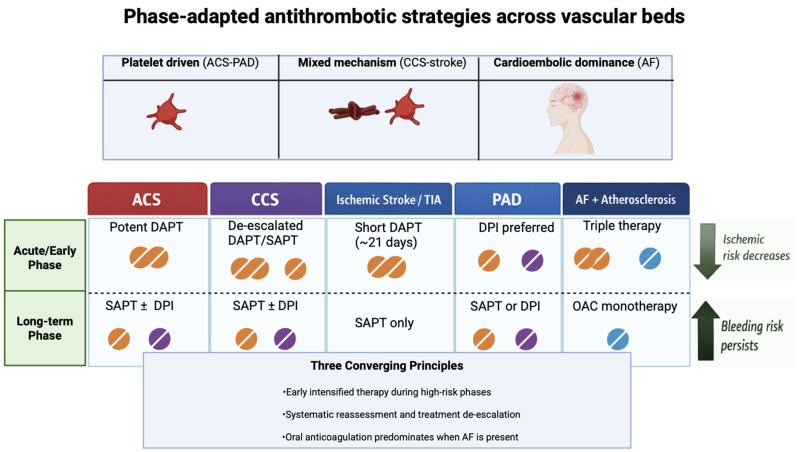
Phase-adapted antithrombotic strategies across vascular beds. This figure summarizes antithrombotic treatment strategies across major atherosclerotic clinical settings, integrating pathophysiological mechanisms and temporal phases of disease. The horizontal axis represents different vascular scenarios (acute coronary syndromes [ACS], chronic coronary syndromes [CCS], ischemic stroke/transient ischemic attack [TIA], peripheral artery disease [PAD], and atrial fibrillation [AF] with coexisting atherosclerotic disease), while the vertical axis distinguishes acute/early and long-term phases. In the acute phase, intensified antithrombotic regimens are generally adopted in selected high-risk settings, followed by progressive simplification over time according to the balance between ischemic and bleeding risk. Platelet-driven mechanisms predominate in ACS and PAD, mixed platelet and coagulation activation characterize CCS and ischemic stroke, whereas cardioembolic mechanisms dominate when AF is present. The figure illustrates the concept of treatment de-escalation from early intensive combinations toward simpler long-term regimens, including single antiplatelet therapy, dual pathway inhibition, or oral anticoagulation monotherapy depending on the clinical context. Legend (color coding of pill icons): orange pills (antiplatelet therapy), purple pills (low-dose factor Xa inhibition; rivaroxaban 2.5 mg twice daily as part of dual pathway inhibition), blue pills (full-dose oral anticoagulation).

Third, structured assessment of ischemic and bleeding risks is crucial to inform choices on regimen strategy, duration, and de-escalation, recognizing that risk scores are aids rather than substitutes for clinical judgment. In this perspective, the concept of “net clinical benefit” has become central to contemporary antithrombotic medicine: it is not enough to reduce ischemic events if this comes at the cost of major bleeding events that impact prognosis, quality of life and adherence. In particular, major bleeding after PCI and/or during DAPT is associated with a significant increase in mortality and subsequent complications, with a persistent prognostic effect over time [31,60]. Consequently, the selection of the regimen and duration should integrate not only the probability of events but also the expected clinical consequences, favoring strategies that maximize the absolute benefit in patients at high ischemic risk while preserving hemorrhagic safety. In addition, clinicians should remain attentive to drug-specific adverse effects beyond bleeding, including dyspnea with ticagrelor, gastrointestinal intolerance with aspirin, and the potential renal or dose-related considerations associated with anticoagulant therapies. Finally, low-dose rivaroxaban plus aspirin has emerged as a new paradigm of dual pathway inhibition for patients with stable but high-risk atherosclerotic disease, particularly in CCS and PAD, whereas its role in stroke and AF populations remains circumscribed [56]. The Figure 1 proposed earlier may help clinicians apply these concepts in daily practice. The main antithrombotic strategies across clinical scenarios are summarized in Table 2.

## 5. Gaps in Evidence

Despite major advances, substantial knowledge gaps persist. Another important limitation of the currently available evidence is the underrepresentation of patients with multimorbidity and polyvascular disease in randomized trials, despite these individuals representing a significant proportion of the real-world population. Patients with overlapping vascular territories—such as combined coronary disease, PAD, and prior stroke—are under-represented in randomized trials, limiting the evidence base for multi-territory, multi-mechanism prevention strategies. Similarly, elderly, frail patients and those with significant comorbidities (renal dysfunction, prior bleeding, malignancy) often have the least robust data despite being at the highest absolute risks for both ischemia and bleeding.

The optimal integration of dual pathway inhibition with emerging agents (including factor XIa inhibitors) is uncertain, particularly in populations at high bleeding risk or with concomitant anticoagulation indications [61,62]. In non-cardioembolic ischemic stroke, there is limited evidence to guide intensified strategies beyond short-term DAPT, and no RCT-supported approach for patients with recurrent events despite guideline-directed therapy. The best antithrombotic regimen for AF patients with complex coronary and peripheral disease, especially beyond one year after revascularization, also remains incompletely defined, with current recommendations largely extrapolated from AF-PCI syndromes and post-ACS studies [20,21,57,63].

## 6. Perspectives

Future antithrombotic strategies will likely be shaped by three major developments. First, precision risk stratification using clinical scores, biomarkers, and imaging may better discriminate between patients who truly benefit from intensified antithrombotic regimens and those in whom de-escalation is preferable, thereby improving the net clinical benefit. Second, novel agents such as factor XI/XIa inhibitors aim to uncouple antithrombotic efficacy from bleeding by selectively targeting contact pathway–mediated thrombosis, with promising early data in stroke and coronary populations that may redefine combinations with APT [61,62].

Third, there is growing interest in dynamic, phase-adapted strategies: higher-intensity therapy during well-defined high-risk windows (early post-ACS, post-revascularization, early post-stroke) followed by systematic de-escalation to monotherapy or lower-intensity regimens, potentially guided by serial risk reassessments or even digital health tools. Pragmatic trials focusing on multimorbid patients and implementation science addressing adherence and real-world bleeding mitigation will be crucial to translating these concepts into routine care. At the same time, integrating approaches based on artificial intelligence and machine learning could enable more accurate and dynamic risk prediction, potentially supporting personalized treatment decisions in complex patients.

## 7. Conclusions

Antithrombotic therapy in atherosclerotic vascular disease is transitioning from uniform regimens to individualized, mechanism- and time-dependent, and risk-adapted strategies. In ACS, potent DAPT with flexible duration remains the cornerstone, while in CCS and PAD, dual pathway inhibition with low-dose rivaroxaban plus aspirin offers additional vascular protection in carefully selected high-risk patients. In ischemic stroke, antiplatelet monotherapy is the long-term gold standard, with short-course DAPT confined to minor events, whereas prevention of AF-related stroke is primarily driven by oral anticoagulation.

In patients with AF and concomitant atherosclerotic events, a short period of triple therapy followed by dual therapy and eventual return to OAC monotherapy represents the prevailing paradigm, illustrating the priority given to cardioembolic prevention while mitigating bleeding. Across all settings, careful balancing of ischemic and bleeding risks, attention to patient preferences, and readiness to de-escalate when appropriate are essential. Ongoing research on novel targets, dual pathway inhibition, and precision risk tools promises further refinement, with the ultimate goal of maximizing protection from recurrent vascular events while minimizing harm from excess bleeding. Therefore, the goal is no longer to identify a single optimal strategy for all patients but rather to define a flexible therapeutic pathway that can be adapted over time according to the evolution of individual risk. The growing availability of new therapeutic options and risk stratification tools offers unprecedented opportunities, but at the same time requires a more informed and personalized approach. Looking ahead, the integration of clinical data, biomarkers and imaging information, together with the development of advanced predictive models, could enable true precision medicine in the field of antithrombotic therapy. However, the application of these concepts in clinical practice will require pragmatic studies and effective implementation strategies, with a particular focus on the most complex and fragile patients, in whom the margin between benefit and risk is narrower. Future research should focus on refining individualized antithrombotic strategies by integrating mechanistic insights, dynamic risk assessment, and emerging therapeutic targets.

## Figures and Tables

**Table 1:** Landmark randomized trials informing antithrombotic strategies across vascular beds.

Setting	Trial	Population	Intervention	Comparator	Key Findings
ACS	PLATO	Acute coronary syndrome	Ticagrelor + ASA	Clopidogrel + ASA	Reduced CV death, MI, stroke
ACS	TRITON-TIMI-38	ACS undergoing PCI	Prasugrel + ASA	Clopidogrel + ASA	Reduced ischemic events, increased bleeding
ACS/PCI	TWILIGHT	High-risk PCI	Ticagrelor monotherapy	DAPT	Reduced bleeding without increased ischemia
ACS	TICO	Acute coronary syndrome	Ticagrelor monotherapy	DAPT	Reduced major bleeding
CCS/PAD	COMPASS	Stable CAD/PAD	Rivaroxaban + ASA	ASA	Reduced MACE with increased non-fatal bleeding
PAD	VOYAGER PAD	PAD post-revascularization	Rivaroxaban + ASA	ASA	Reduced CV and limb events
Stroke/TIA	CHANCE	Minor stroke/TIA	Short-term DAPT	ASA	Reduced early recurrence
Stroke/TIA	POINT	Minor stroke/TIA	DAPT	ASA	Reduced ischemic events, increased bleeding
Stroke	NAVIGATE ESUS	ESUS	Rivaroxaban	ASA	No superiority, increased bleeding
Stroke	RE-SPECT ESUS	ESUS	Dabigatran	ASA	Neutral results
AF + PCI	AUGUSTUS	AF with ACS/PCI	DOAC-based dual therapy	Triple therapy	Reduced bleeding
AF + PCI	RE-DUAL-PCI	AF undergoing PCI	Dabigatran dual therapy	Triple therapy	Reduced bleeding

Abbreviations: ACS, acute coronary syndrome; PCI, percutaneous coronary intervention; ASA, aspirin; DAPT, dual antiplatelet therapy; CAD, coronary artery disease; PAD, peripheral artery disease; TIA, transient ischemic attack; ESUS, embolic stroke of undetermined source; AF, atrial fibrillation; DOAC, direct oral anticoagulant; MI, myocardial infarction; CV, cardiovascular; MACE, major adverse cardiovascular events. Key randomized trials summarized in this table include PLATO [14], TRITON-TIMI 38 [15], TWILIGHT [16], TICO [17], COMPASS [4], VOYAGER PAD [5], CHANCE and POINT trials, NAVIGATE ESUS [18], RE-SPECT ESUS [19], and major AF-PCI trials including AUGUSTUS [20] and RE-DUAL PCI [21].

**Table 2:** Guideline-recommended antithrombotic strategies across major clinical settings.

Clinical Scenario	Default Strategy	Alternative Options	Duration	Key Considerations
ACS (no HBR)	ASA + potent P2Y12 inhibitor	De-escalation or short DAPT	12 months	Balance ischemia vs. bleeding
ACS (HBR)	Short DAPT	Early SAPT or P2Y12 monotherapy	1–6 months	Bleeding risk predominates
CCS	ASA (or clopidogrel)	Dual pathway inhibition	Long-term	Consider ischemic risk
CCS high-risk	Rivaroxaban + ASA	Prolonged DAPT	Long-term	Polyvascular disease
Stroke (non-cardioembolic)	SAPT	Short-term DAPT	21–30 days (DAPT)	Avoid long-term DAPT
Stroke (AF-related)	Oral anticoagulation	—	Long-term	Antiplatelets usually avoided
PAD	SAPT	Rivaroxaban + ASA	Long-term	High limb + CV risk
PAD post-revascularization	Rivaroxaban + ASA	SAPT	Long-term	VOYAGER evidence
AF + ACS/PCI	Triple → dual therapy	OAC alone	Stepwise	Minimize triple therapy duration
AF + CCS	OAC alone	OAC + SAPT (selected)	Long-term	Bleeding risk key driver

Abbreviations: ACS, acute coronary syndrome; CCS, chronic coronary syndrome; PAD, peripheral artery disease; AF, atrial fibrillation; PCI, percutaneous coronary intervention; DAPT, dual antiplatelet therapy; SAPT, single antiplatelet therapy; OAC, oral anticoagulation. The treatment strategies summarized in this table are derived from contemporary international guidelines and major randomized trials, including ESC and ACC/AHA guideline documents [2,22,40,49].

## Data Availability

No new data were created or analyzed in the study. Data sharing is not applicable to this article.

## Abbreviations

The following abbreviations are used in this manuscript:

ACC	American College of Cardiology
ACS	Acute Coronary Syndrome(s)
AF	Atrial Fibrillation
AHA	American Heart Association
APT	Antiplatelet Therapy
CAD	Coronary Artery Disease
CCS	Chronic Coronary Syndrome(s)
CV	Cardiovascular
DAPT	Dual Antiplatelet Therapy
DOAC	Direct Oral Anticoagulant
ESC	European Society of Cardiology
HBR	High Bleeding Risk
MACE	Major Adverse Cardiovascular Events
MACCE	Major Adverse Cardiovascular and Cerebrovascular Events
MALE	Major Adverse Limb Events
MI	Myocardial Infarction
OAC	Oral Anticoagulation
PAD	Peripheral Artery Disease
PCI	Percutaneous Coronary Intervention
RCT	Randomized Controlled Trial
SAPT	Single Antiplatelet Therapy
TIA	Transient Ischemic Attack
